# Global prevalence of asthma-COPD overlap (ACO) in the general population: a systematic review and meta-analysis

**DOI:** 10.1186/s12931-019-1198-4

**Published:** 2019-10-23

**Authors:** Mostafa Hosseini, Amir Almasi-Hashiani, Mahdi Sepidarkish, Saman Maroufizadeh

**Affiliations:** 1 Department of Epidemiology and Biostatistics, School of Public Health, Tehran University of Medical Sciences, Tehran, Iran; 2 Department of Epidemiology, School of Health, Arak University of Medical Sciences, Arak, Iran; 3 Department of Biostatistics and Epidemiology, Babol University of Medical Sciences, Babol, Iran; 4 School of Nursing and Midwifery, Guilan University of Medical Sciences, Rasht, Iran

**Keywords:** Asthma, COPD, Asthma- COPD overlap, Prevalence, Meta-Analysis, Systematic Review

## Abstract

**Background:**

Asthma-COPD overlap (ACO) is a term that encompasses patients with features of both asthma and COPD. To date, the global prevalence of ACO in the general population remains unknown. The objective of this study was to estimate the prevalence of ACO in the general population using a systematic review and meta-analysis.

**Methods:**

A systematic search of ISI Web of Knowledge, MEDLINE/PubMed, and Scopus was performed up to May 2019 to identify studies reporting the prevalence of ACO. Reference lists from identified studies and relevant review articles were also searched. Eligibility criteria were studies reporting the prevalence of ACO, performed in general population, and published in English language. Pooled prevalence of ACO with 95% confidence interval (CI) was calculated using random effects Meta-analysis.

**Results:**

A total of 27 studies were included in this meta-analysis. The Cochran Q test and I^2^ statistics revealed substantial heterogeneity among studies. Based on the random-effects model, the pooled prevalence of ACO was 2.0% (95% CI: 1.4–2.6%) in the general population, 26.5% (95% CI: 19.5–33.6%) among patients with asthma, and 29.6% (95% CI: 19.3–39.9%) among patients with COPD. In addition, for included studies, the global prevalence of asthma-only was 6.2% (95% CI: 5.0–7.4%) and COPD-only was 4.9% (95% CI: 4.3–5.5%).

**Conclusion:**

We estimated the global prevalence of ACO based on population-based studies and found that 2.0% of the general population is affected. However, the prevalence of ACO depends on its diagnostic criteria. Therefore, there is a vital need to better define the ACO diagnostic criteria, management and treatment. It is worth noting that the limitations of the present study include lack of studies in some region of the world and small number of studies included in the subgroup analyses.

## Background

Asthma and chronic obstructive pulmonary disease (COPD) are major public health problem and represent a leading cause of morbidity and mortality worldwide [1, 2]. Asthma and COPD are the most common chronic respiratory diseases worldwide each with a unique natural history and pathophysiology [1]. Asthma is usually characterized by chronic airway inflammation whereas COPD is characterized by persistent respiratory symptoms and chronic inflammation of the airways [3, 4]. However, patients can sometimes have clinical features of both diseases, and this condition has been termed asthma-COPD overlap (ACO), recommended by joint GINA (Global Initiative for Asthma) and GOLD (Global Initiative for Chronic Obstructive Lung Disease) guideline. According to this guideline, ACO is characterized by “persistent airflow limitation with several features usually associated with asthma and several features usually associated with COPD”. Furthermore, many studies use the term asthma-COPD overlap syndrome (ACOS), but based on the recent change recommended by the GINA and GOLD in 2017, we will use ‘ACO’ as a term for this condition as it is not considered a single entity but a group of phenotypes [4]. Although asthma and COPD have been well defined, there is currently no consensus on the definition of ACO.

Most previous studies revealed that patients with ACO have more respiratory symptoms, more frequent exacerbations, poor quality of life, higher mortality rate, increased health care utilization, and higher prevalence of comorbidities than those with either asthma or COPD only [5–9].

Numerous population-based studies have been carried out to estimate the prevalence of ACO throughout the world, especially in the USA and Europe [10–36]. However, there is a considerable variation among the studies. The prevalence of ACO has varied widely in these studies from 0.3 to 5.0% in the general population, from 3.2 to 51.4% in patients with asthma, and from 12.6 to 55.7% in patients with COPD. Although there are currently a limited number of population-based studies in this context, it has been growing in recent years.

The global prevalence of ACO remains unknown, and no systematic review and meta-analysis of population-based studies has yet been conducted. On the other hand, due to the considerable heterogeneity among the reported prevalence of ACO, and its significant public health impact, the exact prevalence of ACO is critical for strategic plan and health policy. We therefore conducted a systematic review and meta-analysis of the published literature to examine this parameter. We examined the prevalence both in the general population and among patients with asthma or COPD to understand better the absolute burden of this condition.

### Methods

This systematic review adheres to the PRISMA (Preferred Reporting Items for Systematic Reviews and Meta-Analyses) checklist [37].

### Search strategy

A systematic literature search was performed in ISI Web of Knowledge, MEDLINE/PubMed, and Scopus databases up to 30 May 2019 to identify studies reporting the prevalence of ACO. The search strategy in ISI Web of Knowledge is outlined in detail in Table 1. The other databases were searched with similar terms. Reference lists from identified studies and relevant review articles were also searched for studies eligible for inclusion.

**Table 1:** Search strategy in database of ISI Web of Knowledge

Set	Query	Results
#1	(epidemiology) *OR* (incidence) *OR* (Prevalence) *OR* (Frequency)	3483569
#2	(“Asthma-chronic obstructive pulmonary disease overlap syndrome”) *OR* (“Asthma and chronic obstructive pulmonary disease overlap syndrome”) *OR* (“asthma-COPD overlap syndrome”) *OR* (“asthma-COPD overlap”) *OR* (“asthma-COPD”) *OR* (“ACOS”) *OR* (“mixed asthma-COPD phenotype”) *OR* (“Asthma combined with COPD”) *OR* (“coexistence of asthma and COPD”) *OR* (“COPD with asthmatic features”) *OR* (“overlap of asthma-COPD”)	1692
#3	(“Pulmonary Disease, Chronic Obstructive”) *OR* (“Chronic Obstructive Pulmonary Disease”) *OR* (“COPD”) *OR* (“asthma”) *OR* (“asthmatic”) *OR* (“Asthmatic Patient”)	237,190
#4	#1 AND #2 AND #3	258

### Outcomes

Our primary outcome of interest was prevalence of ACO in the general population. Secondary outcomes included the prevalence of ACO in patients with asthma and patients with COPD, prevalence of asthma-only, and prevalence of COPD-only. Table S1 presents definitions of asthma, COPD, and ACO in included studies.

### Inclusion and exclusion criteria

The following inclusion criteria were used to select studies: (1) studies reporting the prevalence of ACO, (2) population-based studies, (3) studies published in English language. No time restriction for publication dates was used. The following articles were excluded: (1) studies with no usable data, (2) repeated or overlapping studies, and (3) reviews, meta-analysis, and case reports articles.

### Data extraction and quality assessment

Data were abstracted independently by two authors (SM and AAH), and discrepancies were resolved by consensus. The following study characteristics were extracted: first author’s name, year of publication year, country, region, study name, study period, age, sample size, and prevalence of ACO (point or period prevalence). Study quality was assessed independently by two authors (SM and AAH) utilizing the Joanna Briggs Institute’s critical appraisal checklist for studies reporting prevalence data [38]. This tool includes 9 items each of which is rated as either yes, no, not clear, or not applicable [38].

### Statistical analysis

All meta-analyses were performed using the Stata version 13.0 (Stata Corp, College Station, TX, USA). The Cochran Q test (*P* < 0.10 was considered indicative of significant heterogeneity) and the I^2^ statistic (values of 25, 50, and 75% were considered representing low, medium and high heterogeneity, respectively) were used to assess the heterogeneity between studies [39]. Due to the substantial heterogeneity between studies, pooled prevalence of ACO was calculated using a random-effects model. Potential sources of heterogeneity were explored through meta-regression and subgroup analyses with regard to the study region, year of publication, and study quality. Egger’s test and a visual inspection of funnel plots were used to assess the presence of publication bias [40, 41].

## Results

### Study selection

**Fig. 1:**
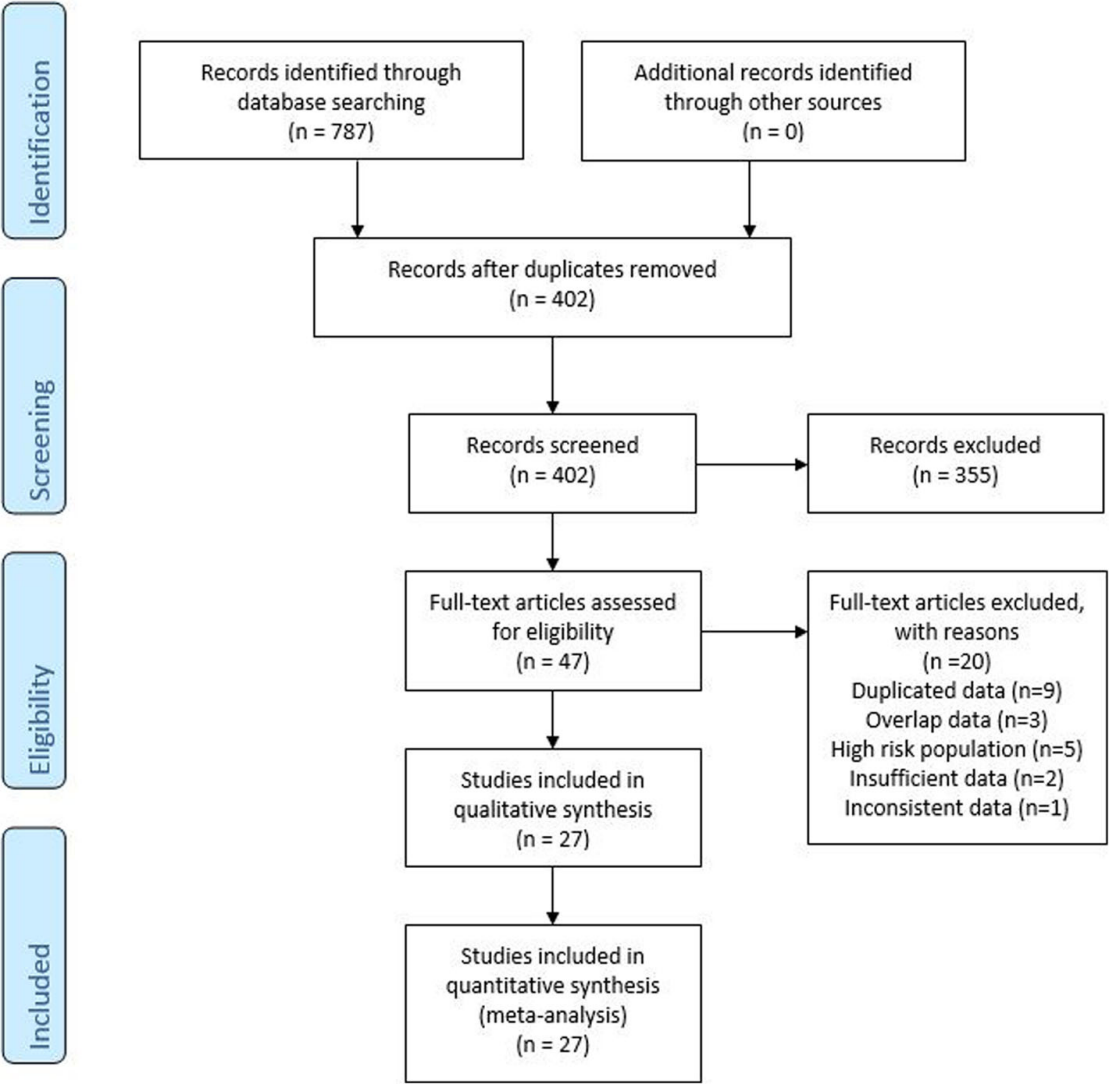
Flow diagram showing search strategy

A flow diagram of the study selection process is presented in Fig. 1. A total of 787 articles were retrieved from the different sources. After removal of duplicates, 402 records remained. By screening titles and abstracts, we identified 47 articles; after the inclusion and exclusion criteria were applied, 27 articles remained.

### Study characteristics

The study characteristics of the included studies are summarized in Table 2. In total, we included 27 studies published since 2011, except for one study that published in 1996: twelve from Europe [10, 12, 13, 16, 17, 20, 23, 25, 28–30, 35], nine from the North America (United States and Canada) [11, 15, 18, 21, 26, 27, 32–34], three East Asia (China and Korea) [19, 22, 36], three from the Latin America and 6 low- and middle-income countries and Australia [14, 24, 31]. The majority of the included studies were published after 2015. As presented in Table S2, the risk of bias was low in the majority of the criteria in the included studies.

**Table 2:** Description of the studies included in the meta-analysis

	First author	Pub. Year	Country	Region	Study	Study Period	Age	Sample Size	Prevalence (%)				
									ACO	Asthma-only	COPD-only	ACO in asthma patients	ACO in COPD Patients
1	Walsh LJ [10]	1996	UK	Europe	NA	NA	> 4	38,865	0.3	8.4	0.8	3.2	25.4
2	Diaz-Guzman E [11]	2011	USA	North America	NHANES III	1988–2006	> 25	15,203	2.3	4.7	5.4	33.5	30.5
3	de Marco R [12]	2013	Italy	Europe	GEIRD	2007- ...	20–84	8360	2.1	6.7	5.2	23.8	28.9
4	Miravitlles M [13]	2013	Spain	Europe	EPI-SCAN	2006–2007	40–80	3802	1.8	NA	8.4	NA	17.4
5	Menezes AMB [14]	2014	Latin America	Latin America	PLATINO	2002–2004	> 40	5044	1.8	1.7	11.8	51.4	13.0
6	Pleasants RA [15]	2014	USA	North America	NC BRFSS	2007–2009	> 18	24,073	3.4	5.2	4.7	39.3	41.4
7	Lindström I [16]	2015	Finland	Europe	NA	2000–2001	30–63	3406	0.7	5.9	2.6	11.1	22.3
8	van Boven JF [17]	2016	Spain	Europe	MAJORICA	2012–2013	> 18	916,843	0.6	NA	2.5	NA	18.3
9	Kumbhare S [18]	2016	USA	North America	BRFSS	2012	> 35	80,498	3.2	5.6	6.0	36.6	35.0
10	Ding B [19]	2016	China	East Asia	China NHWS	2010–2013	> 18	59,935	0.6	1.4	2.7	30.7	18.6
11	Bonten TN [20]	2016	The Netherlands	Europe	NEO	2008–2012	45–65	5675	1.3	NA	NA	NA	NA
12	Mannino DM [21]	2017	USA	North America	NHANES	2007–2012	20–79	12,964	1.9	5.0	11.4	27.0	14.0
13	Kim J [22]	2017	Korea	East Asia	KNHANES IV	2007–2009	> 19	11,656	2.2	5.8	8.4	27.7	20.8
14	Ferrante G [23]	2017	Italy	Europe	PASSI	2013–2015	18–69	108,705	1.0	3.4	2.6	NA	NA
15	Bui DS [24]	2017	Australia	Australia	TAHS	2006–2008	45	1355	5.0	19.9	4.4	20.2	53.5
16	Baarnes CB [25]	2017	Denmark	Europe	DCH	1993–2013	50–64	57,053	1.2	2.1	5.9	35.9	16.4
17	Kendzerska T [26]	2017	Canada	North America	ICES	2002–2012	> 35	7,589,414	3.3	NA	NA	NA	NA
18	Senthilselvan A [27]	2018	Canada	North America	CHMS	2007–2013	> 30	9059	1.6	6.6	2.2	19.3	41.9
19	Henriksen AH [28]	2018	Norway	Europe	HUNT	2006–2008	> 20	50,777	1.9	9.8	1.5	16.2	55.2
20	Ekerljung L [29]	2018	Sweden	Europe	WSAS	2008–2012	16–75	1172	3.4	NA	NA	NA	NA
21	Mindus S [30]	2018	Northern Europe	Europe	GA2LEN and RHINE III	2008 and 2010–2012	> 40	25,429	1.0	6.5	1.4	13.1	40.8
22	Morgan BW [31]	2018	6 low- and middle-income countries		CRONICAS, PRISA, and LiNK	2010- ...	35–92	11,923	3.8	13.6	4.8	21.7	43.8
23	Mendy A [32]	2018	USA	North America	NHNES	2007–2012	> 40	7570	1.0	NA	NA	14.6	12.6
24	Kumbhare S [33]	2018	USA	North America	NHNES III	NA	NA	4434	2.8	2.7	7.7	51.2	27.0
25	Koleade A [34]	2018	Canada	North America	APS	2012	> 12	28,410	2.7	NA	NA	NA	NA
26	Guerriero M [35]	2018	Italy	Europe	NA	2011–2012	20–79	1236	2.1	5.6	9.1	27.4	18.8
27	Kang HR [36]	2019	Korea	East Asia	NHIS–NSC	2003–2011	> 40	1,113,656	1.4		1.1		55.7

ACO: Asthma-COPD Overlap; COPD: Chronic Obstructive Pulmonary Disease

NA: Not available

### Prevalence of ACO in the general population

**Fig. 2:**
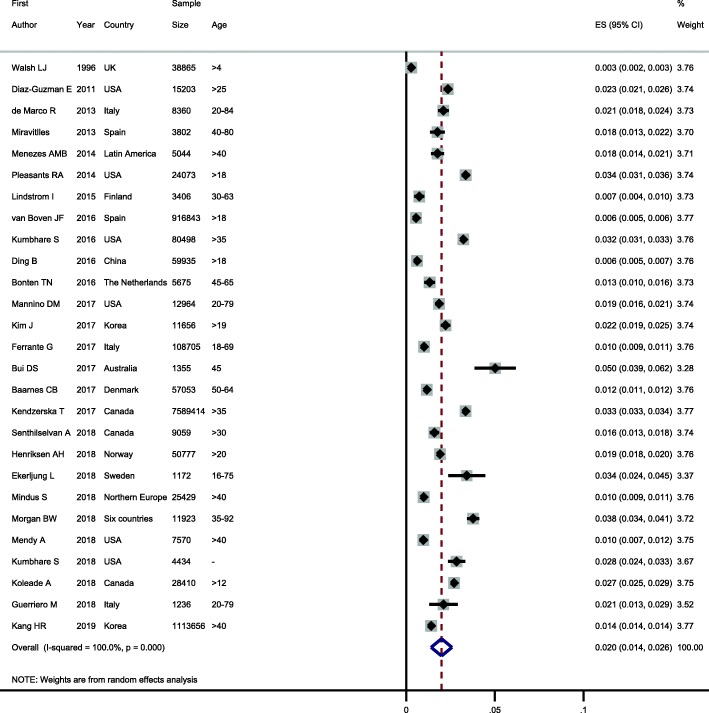
Forest plot showing prevalence of ACO in the general population. Note. Squares represent study-specific prevalence estimates (size of the square denotes the study-specific statistical weight); Horizontal lines represent 95% confidence intervals (CIs); Diamond represents summary estimate of Prevalence with corresponding 95% CI.

**Fig. 3:**
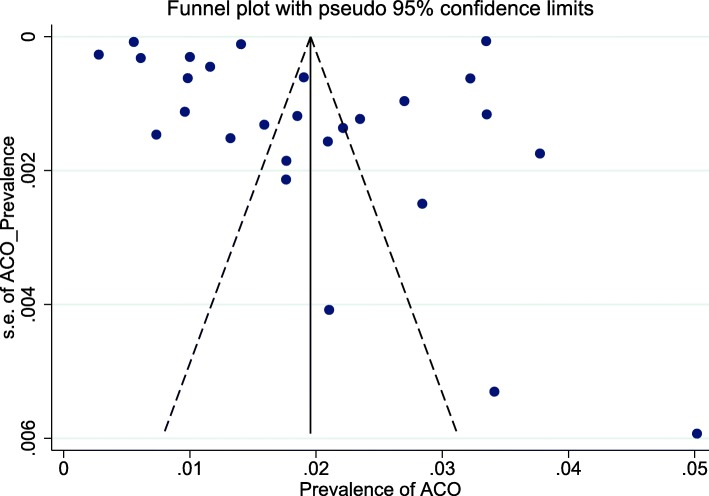
Funnel plot for assessing publication bias in meta-analysis for ACO in general population

As seen in Fig. 2, the lowest and highest prevalence of ACO in the general population was reported by Walsh et al. in UK (0.3%) [10] and Bui et al. in Australia (5.0%) [24], respectively. The Cochran Q test and I^2^ statistics revealed substantial heterogeneity among studies (Q_(26)_ = 88,698.3, *P* < 0.001; I^2^ = 100%). Therefore, random effects model was used for statistical analysis. The pooled prevalence of ACO in the general population was 2.0% (95% CI: 1.4–2.6%) (Table 3). Furthermore, the shape of funnel plots did not reveal any evidence of obvious asymmetry (publication bias) (Fig. 3). Egger’s test also did not reveal any evidence of publication bias (*P* = 0.576).

**Table 3:** Summary of meta-analysis results

Outcomes	Pooled estimates			Heterogeneity		
	NO. of studies	Prevalence (95% CI)	Model	Chi square	P	I square
ACO	27	2.0% (1.4–2.6)	Random	88,698.3	< 0.001	100%
Asthma-only	19	6.2% (5.0–7.4)	Random	9163.5	< 0.001	99.8%
COPD-only	22	4.9% (4.3–5.5)	Random	15,367.2	< 0.001	99.9%
ACO in asthma patients	19	26.5% (19.5–33.6)	Random	17,000,000	< 0.001	100%
ACO in COPD patients	22	29.6% (19.3–39.9)	Random	280,000,000	< 0.001	100%

*ACO* Asthma-COPD Overlap, *COPD* Chronic Obstructive Pulmonary Disease, *CI* Confidence Interval

### Prevalence of asthma-only and COPD-only

For included studies, the global prevalence of asthma-only was 6.2% (95% CI: 5.0–7.4%) and COPD-only was 4.9% (95% CI: 4.3–5.5%).

### Prevalence of ACO among patients with asthma or COPD

**Fig. 4:**
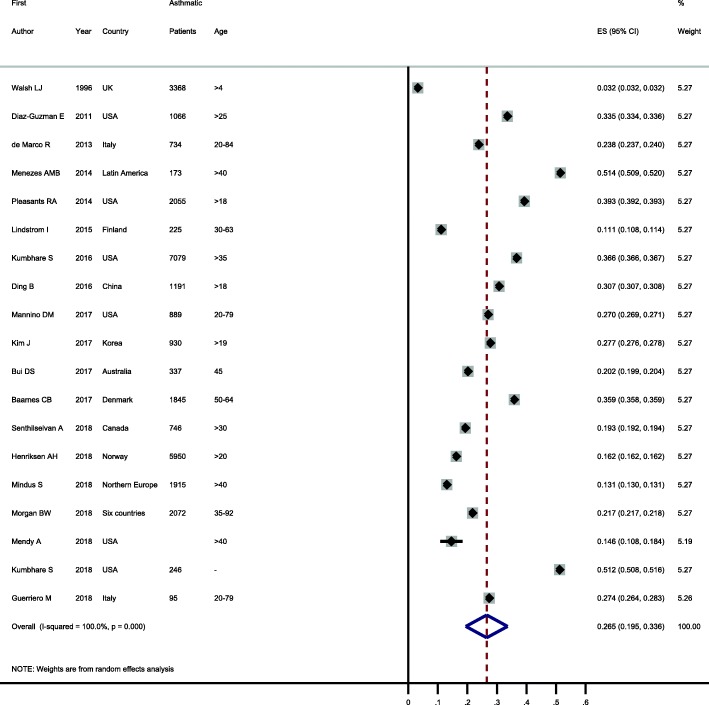
Forest plot showing prevalence of ACO in patients with asthma. Note. Squares represent study-specific prevalence estimates (size of the square denotes the study-specific statistical weight); Horizontal lines represent 95% confidence intervals (CIs); Diamond represents summary estimate of Prevalence with corresponding 95% CI.

**Fig. 5:**
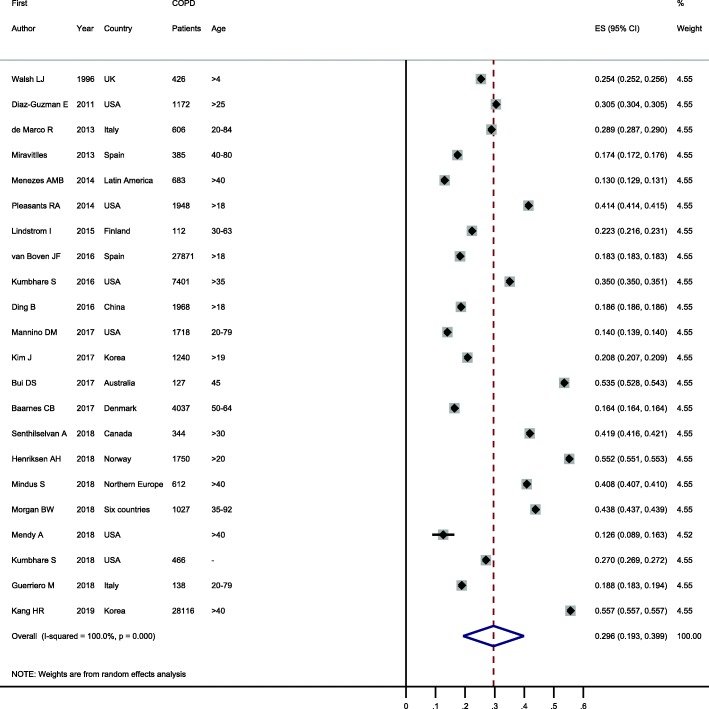
Forest plot showing prevalence of ACO in patients with COPD. Note. Squares represent study-specific prevalence estimates (size of the square denotes the study-specific statistical weight); Horizontal lines represent 95% confidence intervals (CIs); Diamond represents summary estimate of Prevalence with corresponding 95% CI.

Prevalence of ACO among patients with asthma or COPD were reported in 19 and 22 studies, respectively. These values showed considerable variability in patients with asthma (ranging from 3.2 to 51.4%) and COPD (ranging from 13.0 to 55.7%). When these were pooled, the prevalence of ACO was 26.5% (95% CI: 19.5–33.6%) in patients with asthma and 29.6% (95% CI: 19.3–39.9%) in patients with COPD (see Figs. 4 and 5).

### Subgroup analysis

Potential sources of heterogeneity were explored through subgroup analyses with regard to the study region, year of publication (before 2015 vs after 2015), and study quality (≤7 vs ≥8). According to our results, prevalence of ACO was not related to study region (*P* = 0.994), year of publication (*P* = 0.995), and study quality (*P* = 0.865) (Table 4).

**Table 4:** Summary of meta-regression and subgroup analysis for prevalence of ACO in the general population

	NO. of studies	Prevalence of ACO (95% CI)	I-square	Meta-Regression *P*-Value
Region of study				0.994
Europe	12	1.3% (1.0–1.6)	99.1%	
North America	9	2.5% (1.9–3.0)	99.1%	
East Asia	3	1.4% (0.7–2.0)	99.7%	
Year of publication				0.995
≤ 2015	7	1.8% (0.7–2.8)	99.4%	
> 2015	20	2.1% (1.4–2.8)	100.0%	
Study quality				0.865
Low and Moderate (< 8)	5	2.1% (0.7–3.4)	99.5%	
High (≥8)	22	2.0% (1.3–2.6)	100.0%	

*ACO* Asthma-COPD Overlap, *COPD* Chronic Obstructive Pulmonary Disease, *CI* Confidence Interval

## Discussion

ACO is a newly identified condition with apparently high prevalence, and evidently variable features, which is associated with a high burden of disease. Patients with ACO may have worse outcomes than those with either asthma- or COPD-only. Despite its high prevalence, few population-based studies have examined the prevalence of ACO, and as a result, the epidemiology of this condition remains poorly defined. Furthermore, until 2010 only one study has examined the prevalence of ACO in the population-based studies. Here we present a comprehensive overview of the prevalence of ACO in the general population as well as in patients with asthma-only and COPD-only. The overall prevalence of ACO in the general population was 2.0%. Heterogeneity was observed between included studies. This heterogeneity may be explained by differences in the definitions, diagnostic criteria, disease ascertainment methods, geographic region, population characteristics (e.g., age and smoking), study design, and inherent biases associated with observational studies.

Subgroup analysis was performed to assess ACO prevalence in different geographic regions. Most of the studies originated from either North America or Europe, but others came from Latin America, China, Korea, and six low- and middle-income countries. When stratification was performed by geographic region, no differences in ACO prevalence were found. Data were lacking for regions of Africa and Middle East, and Eastern Europe, so we could not examine the prevalence of ACO in these areas. Because data were lacking for regions of Africa and Middle East, and Eastern Europe, ACO estimates in this meta-analysis may overestimate or underestimate the global health burden. Thus, future population-based studies are required to estimate and compare the prevalence of ACO in the different geographic regions. Ideally, we would have investigated whether the year of study contributed significantly to the heterogeneity observed between included studies; however, the considerable overlap of the time periods prevented this analysis. Because of this limitation, we alternatively replace year of study with year of publication and found that ACO prevalence were not changed by year of publication.

Of included studies, only one study reported temporal trend of ACO prevalence, which indicated increasing prevalence in time [26]; however, additional population-based studies are necessary to confirm this finding. This upward trend may be explained by increasing prevalence of both asthma and COPD, and improvement in monitoring of patients. To date, only three studies from Canada, Denmark, and Taiwan examined the incidence of ACO in the general population, emphasizing that such data are missing in the literature. In these studies, the incidence per 1000 person-years was as follows: Canada, 2.1 [26]; Denmark, 0.64 [25]; and Taiwan, 0.70 [42].

The prevalence of ACO among patients with COPD ranged from 12.6 to 55.7% with pooled prevalence of 29.6%. This finding is consistent with a recent meta-analysis, in which the ACO prevalence among patients with COPD in population-based studies was estimated to be 27.0% [8]. In our study, the prevalence of ACO among patients with asthma ranged from 3.2 to 51.4% with pooled prevalence of 26.5%. In previous studies, the prevalence of ACO among patients with asthma also has varied widely from 11.8 to 27.4% [43–47].

For included studies in this review, the global prevalence of asthma-only was 6.2% and COPD-only was 4.9%. In a recent meta-analysis, the global prevalence of COPD based on the GOLD definition was found to be 12.2% [48]. This difference may be due to the different diagnostic criteria and/or including high risk population in the analysis. According to Global Burden of Disease Study 2015, asthma and COPD were the most prevalent chronic respiratory disease, affecting 358 and 174 million people in 2015, respectively. Moreover, COPD and asthma caused 2.6 and 1.1% of global DALYs, respectively [49]. As a large proportion of patients with asthma and COPD have ACO, as well as a worse condition of ACO than asthma- and COPD-only, it is crucial to increase the awareness of ACO and provide clear management and treatment to reduce the disease burden.

This study has several limitations that should be taken into account when interpreting the results. First, there were no data on ACO prevalence for large regions such as Africa and Middle East, and Eastern Europe, thus the generalizability of the results may be limited. Further population-based studies, particularly in Asia and Africa, are required to better estimate the global prevalence and incidence of ACO. Second, regarding the issue that values of ACO prevalence depends on diagnostic methods, we could not perform subgroup analysis for this source of between-study heterogeneity. Third, the quality of the included studies was not optimal. Fourth, the number of studies included in the subgroup analyses was relatively small, so the prevalence of ACO in these subgroups may not be accurately represented. Fifth, although our search was comprehensive, we did not search some other database such as Embase, CINAHL and DOAJ, and include non-English language publications and non-published studies.

## Conclusion

This study showed that 2.0% of the general population is affected by ACO. This finding indicates the considerable clinical impact and major burden posed by ACO. Given that there is no consensus on ACO definition in the literature, and the dependence of its prevalence on diagnostic method, it is important to provide clear diagnostic criteria, management and treatment for this condition.

## Supplementary information

**Additional file 1: Table S1.** Definitions of asthma, COPD, and ACO in included studies. **Table S2.** Quality assessment of included studies in the meta-analysis using Joanna Briggs Institute’s .Critical appraisal checklist for studies reporting prevalence data.

## Data Availability

The datasets used and/or analyzed during the current study are available from the corresponding author on reasonable request.

## Abbreviations

ACO: Asthma-COPD overlap
ACOS: Asthma-COPD overlap syndrome
CI: Confidence Interval
COPD: Chronic obstructive pulmonary disease
GINA: Global Initiative for Asthma
GOLD: Global Initiative for Chronic Obstructive Lung Disease
PRISMA: Preferred Reporting Items for Systematic Reviews and Meta-Analyses

## References

[1] James SL, Abate D, Abate KH, Abay SM, Abbafati C, Abbasi N, Abbastabar H, Abd-Allah F, Abdela J, Abdelalim A (2018). Global, regional, and national incidence, prevalence, and years lived with disability for 354 diseases and injuries for 195 countries and territories, 1990–2017: a systematic analysis for the global burden of disease study 2017. Lancet.

[2] Ehteshami-Afshar S, FitzGerald J, Doyle-Waters M, Sadatsafavi M (2016). The global economic burden of asthma and chronic obstructive pulmonary disease. Int J Tuberc Lung Dis.

[3] Vogelmeier CF, Criner GJ, Martinez FJ, Anzueto A, Barnes PJ, Bourbeau J, Celli BR, Chen R, Decramer M, Fabbri LM. Global strategy for the diagnosis, management, and prevention of chronic obstructive lung disease. 2017 Report. GOLD executive summary. Am J Respir Crit Care Med. 2017;195(5):557–582. 10.1164/rccm.201701-0218PP 28128970

[4] Global Initiative for Asthma (GINA). Global Strategy for Asthma Management and Prevention, Updated 2017. In: www ginasthma org. 2017.

[5] Andersén H, Lampela P, Nevanlinna A, Säynäjäkangas O, Keistinen T (2013). High hospital burden in overlap syndrome of asthma and COPD. Clin Respir J.

[6] Nielsen M, Bårnes CB, Ulrik CS (2015). Clinical characteristics of the asthma–COPD overlap syndrome–a systematic review. Int J Chron Obstruct Pulmon Dis.

[7] Boulet L-P, Hanania NA (2019). The many faces of asthma-chronic obstructive pulmonary disease overlap. Curr Opin Pulm Med.

[8] Alshabanat A, Zafari Z, Albanyan O, Dairi M, FitzGerald J (2015). Asthma and COPD overlap syndrome (ACOS): a systematic review and meta analysis. PLoS One.

[9] Wurst KE, Rheault TR, Edwards L, Tal-Singer R, Agusti A, Vestbo J (2016). A comparison of COPD patients with and without ACOS in the ECLIPSE study. Eur Respir J.

[10] Walsh L, Wong C, S C, Guhan a, Pringle M, Tattersfield A: The prevalence of diagnosed asthma and COPD in a community population in Nottinghamshire *Thorax* 1996, 51:A26.

[11] Diaz-Guzman E, Khosravi M, Mannino DM (2011). Asthma, chronic obstructive pulmonary disease, and mortality in the US population. COPD.

[12] De Marco R, Pesce G, Marcon A, Accordini S, Antonicelli L, Bugiani M, Casali L, Ferrari M, Nicolini G, Panico MG (2013). The coexistence of asthma and chronic obstructive pulmonary disease (COPD): prevalence and risk factors in young, middle-aged and elderly people from the general population. PLoS One.

[13] Miravitlles M, Soriano JB, Ancochea J, Munoz L, Duran-Tauleria E, Sánchez G, Sobradillo V, García-Río F (2013). Characterisation of the overlap COPD–asthma phenotype. Focus on physical activity and health status. Respir Med.

[14] Menezes AMB, de Oca MM, Pérez-Padilla R, Nadeau G, Wehrmeister FC, Lopez-Varela MV, Muiño A, Jardim JRB, Valdivia G, Tálamo C (2014). Increased risk of exacerbation and hospitalization in subjects with an overlap phenotype: COPD-asthma. Chest.

[15] Pleasants RA, Ohar JA, Croft JB, Liu Y, Kraft M, Mannino DM, Donohue JF, Herrick HL (2014). Chronic obstructive pulmonary disease and asthma–patient characteristics and health impairment. COPD.

[16] Lindström I, Luukkonen R, Vasankari T, Kanervisto M, Heliövaara M, Kainu A, Pallasaho P (2015). Asthma, COPD and the risk of disability pension-11 year register-based follow-up study. Eur Respir J.

[17] van Boven JF, Román-Rodríguez M, Palmer JF, Toledo-Pons N, Cosío BG, Soriano JB (2016). Comorbidome, pattern, and impact of asthma-COPD overlap syndrome in real life. Chest.

[18] Kumbhare S, Pleasants R, Ohar JA, Strange C (2016). Characteristics and prevalence of asthma/chronic obstructive pulmonary disease overlap in the United States. Ann Am Thorac Soc.

[19] Ding B, DiBonaventura M, Karlsson N, Ling X: Asthma-chronic obstructive pulmonary disease overlap syndrome in the urban Chinese population: prevalence and disease burden using the 2010**,** 2012, and 2013 China National Health and wellness surveys. Int J Chron Obstruct Pulmon Dis 2016, 11:1139–1150. 10.2147/COPD.S103873 PMC4907484 27354777

[20] Bonten TN, Kasteleyn MJ, de Mutsert R, Hiemstra PS, Rosendaal FR, Chavannes NH, Slats AM, Taube C (2017). Defining asthma–COPD overlap syndrome: a population-based study. Eur Respir J.

[21] Mannino DM, Gan WQ, Wurst K, Davis KJ (2017). Asthma and chronic obstructive pulmonary disease overlap: the effect of definitions on measures of burden. Chronic Obstr Pulm Dis.

[22] Kim J, Kim YS, Kim K, Oh Y-M, Yoo KH, Rhee CK, Lee JH (2017). Socioeconomic impact of asthma, chronic obstructive pulmonary disease and asthma-COPD overlap syndrome. J Thorac Dis.

[23] Ferrante G, Baldissera S, Campostrini S (2017). Epidemiology of chronic respiratory diseases and associated factors in the adult Italian population. Eur J Pub Health.

[24] Bui DS, Burgess JA, Lowe AJ, Perret JL, Lodge CJ, Bui M, Morrison S, Thompson BR, Thomas PS, Giles GG (2017). Childhood lung function predicts adult chronic obstructive pulmonary disease and asthma–chronic obstructive pulmonary disease overlap syndrome. Am J Respir Crit Care Med.

[25] Baarnes CB, Andersen ZJ, Tjønneland A, Ulrik CS (2017). Incidence and long-term outcome of severe asthma–COPD overlap compared to asthma and COPD alone: a 35-year prospective study of 57,053 middle-aged adults. Int J Chron Obstruct Pulmon Dis.

[26] Kendzerska T, Sadatsafavi M, Aaron SD, Lougheed MD, FitzGerald JM, Gershon AS, Network CRR, To TM (2017). Concurrent physician-diagnosed asthma and chronic obstructive pulmonary disease: a population study of prevalence, incidence and mortality. PLoS One.

[27] Senthilselvan A, Beach J. Characteristics of asthma and COPD overlap syndrome (ACOS) in the Canadian population. J Asthma. 2018:1–9. 10.1080/02770903.2018.1531997 30359154

[28] Henriksen AH, Langhammer A, Steinshamn S, Mai X-M, Brumpton BM (2018). The prevalence and symptom profile of asthma–COPD overlap: the HUNT study. COPD.

[29] Ekerljung L, Mincheva R, Hagstad S, Bjerg A, Telg G, Stratelis G, Lötvall J (2018). Prevalence, clinical characteristics and morbidity of the asthma-COPD overlap in a general population sample. J Asthma.

[30] Mindus S, Malinovschi A, Ekerljung L, Forsberg B, Gíslason T, Jõgi R, Franklin KA, Holm M, Johannessen A, Middelveld R (2018). Asthma and COPD overlap (ACO) is related to a high burden of sleep disturbance and respiratory symptoms: results from the RHINE and Swedish GA2LEN surveys. PLoS One.

[31] Morgan BW, Grigsby MR, Siddharthan T, Chowdhury M, Rubinstein A, Gutierrez L, Irazola V, Miranda JJ, Bernabe-Ortiz A, Alam D (2019). Epidemiology and risk factors of asthma-chronic obstructive pulmonary disease overlap in low-and middle-income countries. J Allergy Clin Immunol.

[32] Mendy A, Forno E, Niyonsenga T, Carnahan R, Gasana J (2018). Prevalence and features of asthma-COPD overlap in the United States 2007–2012. Clin Respir J.

[33] Kumbhare S, Strange C (2018). Mortality in asthma-chronic obstructive pulmonary disease overlap in the United States. South Med J.

[34] Koleade A, Farrell J, Mugford G, Gao Z (2018). Prevalence and risk factors of ACO (asthma-COPD overlap) in aboriginal people. J Environ Public Health.

[35] Guerriero M, Caminati M, Viegi G, Senna G, Pomari C (2019). Prevalence and features of asthma–chronic obstructive pulmonary disease overlap in northern Italy general population. J Asthma.

[36] Kang H-R, Hong S-H, Ha S-Y, Kim T-B, Lee E-K (2019). Differences in the risk of mood disorders in patients with asthma-COPD overlap and in patients with COPD alone: a nationwide population-based retrospective cohort study in Korea. Respir Res.

[37] Moher D, Liberati A, Tetzlaff J, Altman DG (2009). Preferred reporting items for systematic reviews and meta-analyses: the PRISMA statement. Ann Intern Med.

[38] Munn Z, Moola S, Lisy K, Riitano D, Tufanaru C (2015). Methodological guidance for systematic reviews of observational epidemiological studies reporting prevalence and cumulative incidence data. Int J Evid Based Healthc.

[39] Higgins J, Thompson SG (2002). Quantifying heterogeneity in a meta-analysis. Stat Med.

[40] Begg CB, Mazumdar M. Operating characteristics of a rank correlation test for publication bias. Biometrics. 1994:1088–101. 7786990

[41] Egger M, Smith GD, Schneider M, Minder C (1997). Bias in meta-analysis detected by a simple, graphical test. BMJ.

[42] Shantakumar S, Pwu R-F, D’Silva L, Wurst K, Kuo Y-W, Yang Y-Y, Juan Y-C, Chan KA (2018). Burden of asthma and COPD overlap (ACO) in Taiwan: a nationwide population-based study. BMC Pulm Med.

[43] Sevimli N, Yapar D, Türktaş H (2019). The prevalence of asthma-COPD overlap (ACO) among patients with asthma. Turk Thorac J.

[44] Kiljander T, Helin T, Venho K, Jaakkola A, Lehtimäki L (2015). Prevalence of asthma–COPD overlap syndrome among primary care asthmatics with a smoking history: a cross-sectional study. NPJ Prim Care Respir Med.

[45] Tommola M, Ilmarinen P, Tuomisto LE, Lehtimäki L, Haanpää J, Niemelä O, Kankaanranta H (2017). Differences between asthma–COPD overlap syndrome and adult-onset asthma. Eur Respir J.

[46] Harada T, Yamasaki A, Fukushima T, Hashimoto K, Takata M, Kodani M, Okazaki R, Takeda K, Watanabe M, Kurai J (2015). Causes of death in patients with asthma and asthma–chronic obstructive pulmonary disease overlap syndrome. Int J Chron Obstruct Pulmon Dis.

[47] Watanabe M, Noma H, Kurai J, Sano H, Ueda Y, Mikami M, Yamamoto H, Tokuyasu H, Kato K, Konishi T (2016). Differences in the effects of asian dust on pulmonary function between adult patients with asthma and those with asthma–chronic obstructive pulmonary disease overlap syndrome. Int J Chron Obstruct Pulmon Dis.

[48] Varmaghani M, Dehghani M, Heidari E, Sharifi F, Moghaddam SS, Farzadfar F (2019). Global prevalence of chronic obstructive pulmonary disease: systematic review and meta-analysis. East Mediterr Health J.

[49] Soriano JB, Abajobir AA, Abate KH, Abera SF, Agrawal A, Ahmed MB, Aichour AN, Aichour I, Aichour MTE, Alam K (2017). Global, regional, and national deaths, prevalence, disability-adjusted life years, and years lived with disability for chronic obstructive pulmonary disease and asthma, 1990–2015: a systematic analysis for the global burden of disease study 2015. Lancet Respir Med.

