# Supplementary material for: Global prevalence of asthma-COPD overlap (ACO) in the general population: a systematic review and meta-analysis
Source: Respir Res. 2019 Oct 23;20:229. doi: 10.1186/s12931-019-1198-4 (PMC6813073; doi:10.1186/s12931-019-1198-4)
Supplement: Supplementary file 1 — Additional file 1: Table S1. Definitions of asthma, COPD, and ACO in included studies. Table S2. Quality assessment of included studies in the meta-analysis using Joanna Briggs Institute’s .Critical appraisal checklist for studies reporting prevalence data. [file 12931_2019_1198_MOESM1_ESM.docx]

**Table S1.** Definitions of asthma, COPD, and ACO in included studies.

|  | First author | Asthma definition | COPD definition | ACO definition |
| --- | --- | --- | --- | --- |
| 1 | Walsh LJ [10] | Not available, abstract | Not available, abstract | Not available, abstract |
| 2 | Diaz-Guzman E [11] | Self-reported physician diagnosis of asthma | Self-reported physician diagnosis of chronic bronchitis or emphysema | Combination of asthma and COPD |
| 3 | de Marco R [12] | Self-reported physician diagnosis of asthma | Self-reported physician diagnosis of chronic bronchitis, COPD or emphysema | Combination of asthma and COPD |
| 4 | Miravitlles M [13] | Self-reported physician diagnosis of asthma | Post-bronchodilator FEV_1_/FVC <0.70. | Combination of asthma and COPD |
| 5 | Menezes AMB [14] | Report of wheezing in the last 12 months and  post-bronchodilator increase in FEV1 or FVC of 200 mL and 12% | Post-bronchodilator FEV_1_/FVC <0.70. | Combination of asthma and COPD |
| 6 | Pleasants RA [15] | Self-reported physician diagnosis of asthma | Self-reported physician diagnosis of COPD, chronic bronchitis or emphysema | Combination of asthma and COPD |
| 7 | Lindström I [16] | Self-reported physician diagnosis of asthma | Post-bronchodilator FEV_1_/FVC ratio of <0.70. | Having both asthma and COPD |
| 8 | [van Boven JF](https://www.ncbi.nlm.nih.gov/pubmed/?term=van%20Boven%20JF%5BAuthor%5D&cauthor=true&cauthor_uid=26836892) [17] | Physician-confirmed diagnosis of asthma (ICD-9 code: 493) | Physician-confirmed diagnosis of asthma (ICD-9 codes: 491, 492 and/or 496) | Physician-confirmed diagnosis of both asthma and COPD |
| 9 | Kumbhare S [18] | Self-reported physician diagnosis of asthma | Self-reported physician diagnosis of COPD, chronic bronchitis or emphysema | Self-reported physician diagnosis of both asthma and COPD |
| 10 | Ding B [19] | Self-reported physician diagnosis of asthma | Self-reported physician diagnosis of COPD, chronic bronchitis or emphysema | Self-reported physician diagnosis of both asthma and COPD |
| 11 | Bonten TN [20] | Self-reporting in baseline questionnaires or by ICPC codes in the primary care EMR (code R96). | Self-reporting in baseline questionnaires or by ICPC codes in the primary care EMR (code R95). | Presence of Asthma and COPD in the EMR, questionnaires, and lung function (FEV_1_/FVC and FeNO), or a combination of these. |
| 12 | Mannino DM [21] | Self-reported physician diagnosis of asthma | Post-bronchodilator FEV_1_/FVC <0.70. | Post-bronchodilator FEV_1_/FVC <0.70 and current asthma. |
| 13 | Kim J [22] | experiencing wheezing during the twelve months prior to the survey. | Post-bronchodilator FEV_1_/FVC <0.70. | having FEV_1_/FVC <0.7 and experiencing wheezing during the twelve months prior to the survey |
| 14 | Ferrante G [23] | Self-reported physician diagnosis of asthma | Self-reported physician diagnosis of chronic  bronchitis, emphysema or respiratory failure (COPD) | Self-reported physician diagnosis of both asthma and COPD |
| 15 | Bui DS [24] | Having a positive response to the question  “have you ever had asthma?” plus any asthma symptom or asthma medication use in the last 12 months. | Post- bronchodilator FEV_1_/FVC less than the Global Lung Initiative lower limit of normal. | Combination of asthma and COPD |
| 16 | Baarnes CB [25] | ICD-10 codes DJ45–46 | ICD-10 codes DJ40–44 | Having at least one hospital admission for asthma together with at least one admission for COPD |
| 17 | Kendzerska T [26] | Physician-confirmed diagnosis of asthma | Physician-confirmed diagnosis of COPD | Physician-confirmed diagnosis of both asthma and COPD |
| 18 | Senthilselvan A [27] | Self-reported physician diagnosis of asthma | Self-reported physician diagnosis of COPD, chronic bronchitis or emphysema | Self-reported physician diagnosis of both asthma and COPD |
| 19 | Henriksen AH [28] | Self-reported physician diagnosis of asthma | Pre-bronchodilator FEV_1_/FVC <0.70. | Self-reported physician diagnosis of asthma and pre- bronchodilator FEV1_/_FVC < 0.70. |
| 20 | Ekerljung L [29] | fulfilling at least one of 4 criteria:  1) reporting a physician-diagnosed asthma with respiratory symptoms or use of asthma medication during the last 12 months, 2) a positive reversibility test with an increase in FEV_1_ of >12% and >200 ml, 3) reporting ever asthma, and a positive methacholine challenge with asthma symptoms during the last 12 months or 4) reporting ever asthma and asthma symptoms during the last 12 months with increased blood eosinophils | Post-bronchodilator FEV_1_/FVC <0.70. | having a post-bronchodilator FEV_1_/FVC<0.7; in addition, subjects should fulfil at least 1 of 4 criteria for asthma |
| 21 | Mindus S [30] | Positive answer to either the question `Have you had an attack of asthma in the last 12 months?' or the question `Are you currently taking any medicine, including  inhalers, aerosols or tablets, for asthma?' | Self-reported physician diagnosis of COPD | Combination of asthma and COPD |
| 22 | Morgan BW [31] | fulfilling 1 of 3 criteria: self-report of wheezing in 1 year, self-report of medication use for asthma in 1 year, or self-report of a physician diagnosis of asthma | Postbronchodilator FEV1/FVC ratio below the lower limit of normal (defined as the lowest 5th percentile) of the Global Lung Function Initiative mixed-ethnic reference population | Presence of both asthma and COPD |
| 23 | Mendy A [32] | Self-reported physician diagnosis of asthma | Post-bronchodilator FEV_1_/FVC <0.70. | Co-existence of both asthma and COPD |
| 24 | Kumbhare S [33] | Not available, abstract | Not available, abstract | Not available, abstract |
| 25 | Koleade A [34] | Self-reported physician diagnosis of asthma | Self-reported physician diagnosis of COPD, chronic bronchitis or emphysema | Self-reported physician diagnosis of both asthma and COPD |
| 26 | Guerriero M [35] | Not available | Not available | airway obstruction (FEV1/ FVC below the lower limit of normal; a highly positive BD test (increase in FEV_1_≥15% and FVC≥400 mL); and self-reported physician diagnosis of asthma and atopy |
| 27 | Kang HR [36] | diagnosis with asthma at least twice, as a principal or secondary diagnosis (ICD-10 codes J45 and J46) and use of COPD medication | Diagnosis with COPD at least twice as a principal or secondary diagnosis (ICD-10 codes J42, J43 and J44) and use of COPD medication | Meeting both criteria for COPD  and asthma |

ACO: Asthma-COPD Overlap; COPD: Chronic Obstructive Pulmonary Disease

**Table S2**. Quality assessment of included studies in the meta-analysis using Joanna Briggs Institute’s critical appraisal checklist for studies reporting prevalence data

|  |  | 1 | 2 | 3 | 4 | 5 | 6 | 7 | 8 | 9 | Quality Score |
| --- | --- | --- | --- | --- | --- | --- | --- | --- | --- | --- | --- |
|  |  | Was the sample frame appropriate to address the target population? | Were study participants sampled in an appropriate way? | Was the sample size adequate? | Were the study subjects and the setting described in detail? | Was the data analysis conducted with sufficient coverage of the identified sample? | Were valid methods used for the identification of the condition? | Was the condition measured in a standard, reliable way for all participants? | Was there appropriate statistical analysis? | Was the response rate adequate, and if not, was the low response rate managed appropriately? |  |
| 1 | Walsh LJ [10] | Y | Y | Y | Y | Y | U | U | Y | U | 6 |
| 2 | Diaz-Guzman E [11] | Y | Y | Y | Y | Y | Y | Y | Y | U | 8 |
| 3 | de Marco R [12] | Y | Y | Y | Y | Y | Y | Y | Y | U | 8 |
| 4 | Miravitlles M [13] | Y | Y | Y | Y | Y | Y | Y | Y | Y | 9 |
| 5 | Menezes AMB [14] | Y | Y | Y | Y | Y | Y | Y | Y | U | 8 |
| 6 | Pleasants RA [15] | Y | Y | Y | Y | Y | Y | Y | Y | Y | 9 |
| 7 | Lindström I [16] | Y | Y | Y | Y | Y | Y | Y | Y | U | 8 |
| 8 | [van Boven JF](https://www.ncbi.nlm.nih.gov/pubmed/?term=van%20Boven%20JF%5BAuthor%5D&cauthor=true&cauthor_uid=26836892) [17] | Y | Y | Y | Y | Y | Y | Y | Y | U | 8 |
| 9 | Kumbhare S [18] | Y | Y | Y | Y | Y | Y | Y | Y | Y | 9 |
| 10 | Ding B [19] | Y | Y | Y | Y | Y | Y | Y | Y | N | 8 |
| 11 | Bonten TN [20] | Y | N | Y | N | Y | Y | Y | N | Y | 6 |
| 12 | Mannino DM [21] | Y | Y | Y | Y | Y | Y | Y | Y | Y | 9 |
| 13 | Kim J [22] | Y | Y | Y | Y | Y | Y | Y | Y | N | 8 |
| 14 | Ferrante G [23] | Y | Y | Y | Y | Y | Y | Y | Y | N | 8 |
| 15 | Bui DS [24] | Y | Y | Y | Y | Y | Y | Y | Y | N | 8 |
| 16 | Baarnes CB [25] | Y | Y | Y | Y | Y | Y | Y | Y | N | 8 |
| 17 | Kendzerska T [26] | Y | Y | Y | Y | Y | Y | Y | Y | U | 8 |
| 18 | Senthilselvan A [27] | Y | Y | Y | Y | Y | Y | Y | Y | U | 8 |
| 19 | Henriksen AH [28] | Y | Y | Y | Y | Y | Y | Y | Y | N | 8 |
| 20 | Ekerljung L [29] | Y | Y | Y | Y | Y | Y | Y | N | N | 7 |
| 21 | Mindus S [30] | Y | Y | Y | Y | Y | Y | Y | Y | N | 8 |
| 22 | Morgan BW [31] | Y | Y | Y | Y | Y | Y | Y | Y | Y | 9 |
| 23 | Mendy A [32] | Y | Y | Y | Y | Y | Y | Y | Y | U | 8 |
| 24 | Kumbhare S [33] | Y | Y | Y | U | Y | U | U | Y | U | 5 |
| 25 | Koleade A [34] | Y | Y | Y | Y | Y | Y | Y | N | U | 7 |
| 26 | Guerriero M [35] | Y | Y | Y | Y | Y | Y | Y | Y | U | 8 |
| 27 | Kang HR [36] | Y | Y | Y | Y | Y | Y | Y | Y | U | 8 |

Y: Yes; N: No; U=Unclear; NA: Not applicable.
